# Impaired coherence of life narratives of patients with schizophrenia

**DOI:** 10.1038/srep12934

**Published:** 2015-08-10

**Authors:** Mélissa C. Allé, Jevita Potheegadoo, Christin Köber, Priscille Schneider, Romain Coutelle, Tilmann Habermas, Jean-Marie Danion, Fabrice Berna

**Affiliations:** 1 INSERM U-1114, 1 place de l’Hôpital, Clinique Psychiatrique, Strasbourg, France; 2 Université de Strasbourg, Faculté de Médecine, 4 rue Kirchleger, Strasbourg, France; 3 FMTS: Fédération de Médecine Translationnelle de Strasbourg, France; Fondation FondaMental, Créteil, France; 4 Hôpitaux Universitaires de Strasbourg, 1 place de l’Hôpital, Strasbourg, France; 5 Department of Psychology, Goethe University, Frankfurt am Main, Germany; 6 Centre Hospitalier de Rouffach, Centre de Ressources Autisme Alsace, Rouffach, France; 7 Centre Psychothérapique de Nancy, Centre de Ressources Autisme de Lorraine, 1 rue du Dr Archambault, Laxou, France

## Abstract

Self-narratives of patients have received increasing interest in schizophrenia since they offer unique material to study patients’ subjective experience related to their illness, in particular the alteration of self that accompanies schizophrenia. In this study, we investigated the life narratives and the ability to integrate and bind memories of personal events into a coherent narrative in 27 patients with schizophrenia and 26 controls. Four aspects of life narratives were analyzed: coherence with cultural concept of biography, temporal coherence, causal-motivational coherence and thematic coherence. Results showed that in patients cultural biographical knowledge is preserved, whereas temporal coherence is partially impaired. Furthermore, causal-motivational and thematic coherence are significantly impaired: patients have difficulties explaining how events have modeled their identity, and integrating different events along thematic lines. Impairment of global causal-motivational and thematic coherence was significantly correlated with patients’ executive dysfunction, suggesting that cognitive impairment observed in patients could affect their ability to construct a coherent narrative of their life by binding important events to their self. This study provides new understanding of the cognitive deficits underlying self-disorders in patients with schizophrenia. Our findings suggest the potential usefulness of developing new therapeutic interventions to improve autobiographical reasoning skills.

Disorders of self in patients with schizophrenia are probably one of the most intriguing symptoms of the illness and certainly remain one of most difficult to describe and understand. These disorders have been long considered as defining schizophrenia^1^,^2^, or as representing “the core feature” of schizophrenia^3^,^4^. The concept of self is however complex and ranges from very basic processes anchored in neurobiological processes to more social or transcendental aspects. Therefore, models are needed to circumscribe the research to particular aspects of the concept of self. One of the most influential models of the self has been developed by Gallagher^5^ and proposes the distinction between two complementary dimensions of the self: a minimal and a narrative self. The minimal self consists in a pre-reflexive dimension of the self that supports an immediate “sense of self”. The narrative self corresponds to the dimension of self that is extended in time and provides a sense of continuity and coherence to the self across time^6^,^7^. It is intimately linked to the stories we tell about ourselves and it grounds on the memory of our personal past, called autobiographical memory. This form of memory is essential for the self and is conceived as the “memory of the self” since it groups together various type of information that all relate to the self^8^.

The clinical and experimental literature devoted to self-disorders in schizophrenia has mainly considered aspects of the minimal self. With regard to the narrative dimension of the self, self-narratives of patients have recently received increasing interest in schizophrenia research. Indeed, these narratives offer unique material for figuring out the kind of subjective experience patients have during the course of their illness, in particular the alteration of self which accompanies schizophrenia^9^,^10^. These abnormalities have been extensively described in clinical reports and qualitative research work on schizophrenia, albeit not clearly objectified in experimental research^11^,^12^,^13^. The analysis of first-person accounts has revealed that the lack of coherence in patients’ life is often mirrored in the disorganization of patients’ narratives of their life. Recent research on patients’ self-narratives has benefited from the empirical approaches developed in the domain of autobiographical memory. Several studies have thus shown that autobiographical memory is impaired in schizophrenia and that patients’ self is supported by less vivid, less specific and less organized memories^14^,^15^,^16^,^17^,^18^,^19^,^20^,^21^,^22^. Moreover, patients have difficulties to interpret and give meaning to significant personal events^23^,^24^. Lack of autobiographical reasoning skills was correlated with patients’ executive dysfunction and negative symptoms^23^ and was suggested to account for the reduced coherence of their narratives^24^. These results lead to the hypothesis that executive dysfunction and autobiographical reasoning impairment may represent cognitive mechanisms explaining self-disorders in schizophrenia^23^.

To date, studies have mostly focused on memories of single personal events. However, impairment of single memories does not allow for assumptions to be made about alterations in coherence and self-continuity in patients with schizophrenia. Thus, investigating the life story as represented by entire life narratives appears to be the most relevant approach to this question. Indeed, mature life narratives coherently organize multiple single event narratives within the context of personal development^6^,^25^. Furthermore, the life narrative has been considered the best suited format for ego identity, because it serves to create a sense of personal coherence, unity, continuity and purpose in one’ life across change. This is of prime importance for mental health and well-being^6^,^25^.

The ability to create a coherent narrative of one’s life emerges only during adolescence, a critical time for identity formation^25^,^26^. According to Habermas and Bluck^26^, the global coherence of the life story has four major aspects (Fig. 1). A first aspect is coherence with a cultural concept of biography, at its core the cultural life script^27^ which defines normative transitional life events and their normative timing (for instance, get a job at 22 years old, get married at 25 years old or have the first child at 27 years old). A second aspect is temporal coherence that reflects narrator’s ability to identify when and in what order events took place and that allows the listener to understand the chronology of the life narrative. In this paper, these two aspects were termed narrative framework, as they serve to form the skeleton of the life story, allowing a sequence order of major events. The two other aspects of global coherence are causal-motivational and thematic coherence. They involve complementary processes that unify diversified experiences, thereby maintaining the sense of self-continuity^28^. On the one hand, causal-motivational coherence enables listeners to understand the narrator’s personal development. Within the narrative, causal-motivational coherence is supported by arguments covering personal change and specifying how events cause other events and influence one’s personality evolution (for instances, “That journey changed many things for me; in that moment I understood what is meant by the meaning of life, and since then I am a little more self-confident”; “When I came back to Vietnam, I realized that in the meantime I had grown away from my own culture, the Vietnamese way of life, let’s say from these Vietnamese traditional mentalities”). On the other hand, thematic coherence enables listeners to apprehend what is stable in narrator’s life across dominant life themes or stable personality traits. This stability is expressed using arguments which explain an action by an enduring personality trait, declare an action as reflecting a trait or clarify in what an event contradicts personality (for instance, “In puberty I was always extremely shy and well-behaved. I mean, I never rebelled against anything. So I was very restricted and limited in my ideas and possibilities. That’s why I had never a boyfriend.”; “Normally, I and the guys in my class, we are really uncool, I mean very well-behaving the whole time. But on that school trip, we freaked out. Oh man, I was so drunk.”). Both types of arguments correspond to the concept of autobiographical reasoning and make it possible to compensate personal discontinuity by embedding autobiographical disruptions and changes in a coherent life story^28^. Hence, global coherence is a property of the entire life narrative and can be measured by ratings (i.e. global ratings of coherence). Specific local text elements contribute to temporal, thematic and causal-motivational global coherence^26^,^29^,^30^ and can be counted in life narratives (i.e. local indicators of coherence).

In this study we investigated possible impairments in the narrative framework and in causal-motivational and thematic coherence of life narratives of patients with schizophrenia. Indeed, the life story framework is highly relevant for understanding better the cognitive mechanisms underlying the lack of personal coherence clinically described in patients with schizophrenia. Based on the above-mentioned studies, we expected to find specific impairment of autobiographical reasoning and consequently of causal-motivational and thematic coherence of patients’ life narratives, which in turn may be related to executive dysfunction^23^,^24^ and a deficit of general self-representation^12^,^17^. We did not make predictions regarding temporal coherence, considering the contradictory results of previous studies showing either a preserved temporal organization of autobiographical memory clusters^14^,^19^ or a reduced chronological coherence within memories of patients with schizophrenia^24^.

## Methods

### Participants

Twenty-seven stabilized outpatients (11 women) were recruited from the Psychiatric Department of Strasbourg’s University Hospital, all fulfilling the DSM-IV-TR diagnosis of schizophrenia^31^. Patients with a major depressive episode (Calgary Depression Scale for Schizophrenia, CDSS^32^ score higher than 6) and patients with an IQ score below 70 (Wechsler Adult Intelligence Scale Revised, WAIS-R^33^) were excluded. All patients except one were receiving long-term neuroleptic treatment (first generation, n = 22; second generation, n = 2; both, n = 2). Nine patients were treated with benzodiazepines and 4 with antiparkinsonian medication. The control group included 26 healthy participants (11 women). Patients and controls had neither current substance abuse nor a history of traumatic brain-injury, epilepsy, or other neurological disorders. Our groups did not differ significantly in terms of age, level of schooling, or IQ.

The IRB of Lille III approved this study and all experiments were conducted in accordance with approved guidelines and regulations. All participants gave their informed written consent.

### Procedure

#### Clinical and neuropsychological assessments

Symptom severity of schizophrenia in patients was assessed using the Positive And Negative Syndrome Scale (PANSS)^34^. All patients were clinically assessed for depression, with the CDSS^32^, and controls completed the Beck Depression Inventory (BDI)^35^. Participants’ anxiety level and self-esteem were assessed by the State-Trait Anxiety Inventory (STAI)^36^ and the Rosenberg Self-Esteem (RSE) Scale^37^ respectively.

Executive functioning, particularly mental flexibility and strategic retrieval of information in memory, was assessed with (1) the Trail-Making Test (TMT, Part A and B)^38^ and (2) the semantic and phonologic verbal fluency tasks^39^. We selected executive functions considered to be particularly involved in life narration and autobiographical reasoning abilities^23^,^40^.

#### Biographical practices

Given that the frequency of biographical practices can influence the construction of the life narrative, they were assessed by asking for frequency ratings of biographical activities^29^ (such as keeping a diary, looking at old pictures, talking about problems with friends).

#### Subjective Sense of Coherence

Participants rated their subjective sense of coherence on the scale developed by Antonovsky^41^ which comprises three subscales: comprehensibility of external events, their manageability, and the meaningfulness ensuing from them. This sense of coherence is a salutogenic index reflecting individual resilience.

#### Life narratives

The protocol developed by Habermas and de Silveira^29^ was used. Participants were asked to recall the seven most important events they had ever experienced and to write them down on cards (see Supplementary Material S1). Then, participants narrated their life story in 20 minutes. Instructions aimed at encouraging participants to orally narrate specific memories, integrating them in the narrative, and explaining how they had become the person they are today (see Supplementary Material S2). The experimenter did not interrupt participants but encouraged them to pursue the narrative if they had time left. All life narratives were audio-recorded.

Afterwards, participants dated the seven memories, rated their vividness and their emotional valence and intensity on 7-point scales. Finally, participants completed the short version of the Centrality of Event Scale (CES)^42^, assessing the extent to which each of the seven events had become a reference point for personal identity or a turning point in their life.

#### Cultural Life Script

To assess knowledge of the Cultural Life Script, participants imagined the seven most important events they thought would happen in a newborn’s life across the life span^27^ and estimated the typical age for each event.

### Scoring

#### Segmentation

After a verbatim transcription, narratives were divided into propositions, which correspond to minimal meaning sentences (see Supplementary Material S3, for examples). Two independent coders segmented 16 life stories into propositions and had an agreement of 93%. Each disagreement was resolved by discussion, remaining narratives were segmented by one of the two coders.

#### Coding

The causal-motivational and thematic coherence as well as the narrative framework were measured at the global and local level, using manuals developed by Habermas and de Silveira^29^ (see Supplementary Material S4 and S5). At the local level, the proportion of indicators of causal-motivational, thematic and temporal coherence was calculated by dividing the number of each category of indicators by the total number of propositions. At the global level, causal-motivational, thematic and temporal coherence of the entire narratives were rated using three different 7-point scales.

#### Causal-motivational and thematic coherence

At the local level, indicators of causal-motivational coherence encompass self-event connections generating identity changes, and other autobiographical arguments relating to behavioral and personality changes. They indicate that the narrator tries to maintain self-continuity despite the occurrence of identity changes. Local indicators of thematic coherence include self-event connections reflecting the stability of identity (e.g. “I’ve always been a shy person and had problems to make friends”). Both causal-motivational and thematic local indicators correspond to autobiographical reasoning.

At the global level, the scale for global causal-motivational coherence of life narratives measures how well they portray the development of the narrator’s personality, whereas the scale for global thematic coherence measures the presence of both a guiding thread and thematic connections across different life episodes^29^,^30^.

All narratives were coded or rated by two independent raters with good inter-rater reliability for both local indicators (causal: ĸ = 0.80; thematic: ĸ = 0.71) and global coherence scales (causal-motivational: r_ic_ = .85; thematic: r_ic_ = .73).

#### Narrative framework

##### Temporal coherence.

We used temporal indicators of date, age, life period, and distance from the present as local indicators of temporal coherence. They allow the reader to locate events across the course of the story.

Moreover, the linear temporal order was also assessed through the proportion of anachronies present in narratives. Every deviation from the linear temporal order encompassing at least four propositions was counted as an anachrony^43^.

Finally, the global temporal coherence was assessed using a 7-point scale measuring how the narrator was able to identify when and in what order events took place.

Inter-rater reliability between the two independent raters was good for local indicators (ĸ = 0.85), anachronies (ĸ = 0.74) and the global coherence scale (r_ic_ = .89).

Scoring of the Cultural Life Script. A typicality score was calculated from the 7 events chosen by the participants, by weighing each nomination by its relative frequency in the normative sample^27^.

### Statistical Analysis

Between-group comparisons of clinical and neuropsychological data were performed using separate one-way analyses of variance (ANOVAs). Separate MANOVAs were performed on the proportions of local indicators and on global scales of causal-motivational and thematic coherences. Concerning temporal coherence, given that years of schooling was significantly correlated with both the proportion of local indicators and global ratings of temporal coherence, separate ANCOVAs were performed on both measures with the number of years of schooling as a covariate. The Cultural Life Script score was analyzed with an ANOVA. Finally, Pearson correlations were calculated between the proportions of local indicators of coherence and the ratings of global coherence on one side, and both clinical and neuropsychological data on the other side.

## Results

### Clinical and neuropsychological measures

Both state and trait anxiety level were higher in the patient group compared to the control group (*p*s < .03), whereas self-esteem was lower in patients than in controls (*p* < .001). Patients’ executive functioning performances were significantly lower than those of controls in both the verbal fluency task (*p* = .002) and the TMT shifting score (TMT B-A) (*p* = .02), taking into consideration that neuropsychological data were missing for two patients (Table 1).

### Biographical practices

Biographical practices did not differ between groups.

### Subjective Sense of Coherence

Patients displayed a significantly lower sense of coherence than controls in all the three subscales (all *p*s < .001). This indicates that patients have difficulties to deal with stressful situations and to preserve a sense of coherence in spite of them.

### Characteristics of autobiographical memories and life narratives

The characteristics of autobiographical memories selected to structure the life narratives did not differ between patients and controls in their vividness, or centrality score (*ps* > .052). Further analyses of CES items revealed that events chosen by patients were neither more disruptive (items 5 and 7) nor less integrated (items 1 to 4) than those of controls (*ps *> .14). Only the mean emotional valence of memories was less positive in patients (*p* = .004). The average time taken to narrate the life story did not differ between groups (*p* = .59), but the patients’ narratives were significantly shorter (*p* = .04) (see Supplementary Material S6).

#### Causal-motivational and thematic coherence

**Figure 2:**
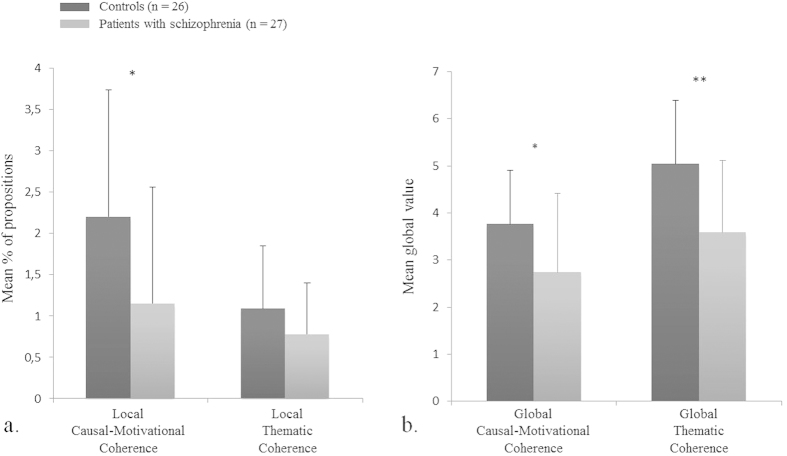
Causal-motivational and thematic coherence: proportion of local indicators and global ratings in patients with schizophrenia and controls. *p < .05; **p < .01.

A MANOVA revealed that autobiographical reasoning (as reflected by both causal-motivational and thematic local indicators) was significantly lower in the patients compared to controls (F(1,51) = 9.57; *p* = .003; ŋ^2^ = .16). Univariate analyses showed that indicators of causal-motivational coherence were significantly reduced in patients’ narratives (F(1,51) = 6.72; *p* = .01; ŋ^2^ = .12), whereas indicators of thematic coherence did not differ between groups (F(1,51) = 2.60; *p* = .11; ŋ^2^ = .05) (Fig. 2).

A MANOVA revealed that global causal-motivational and thematic coherence were significantly lower in the patients than in controls (F(1,51) = 12.45; *p* < .001; ŋ^2^ = .20). Univariate analyses showed that both scores were significantly lower in patients (F(1,51) = 8.85; *p* < .01; ŋ^2^ = .15 and F(1,51) = 6.63; *p* = .01; ŋ^2^ = .12 respectively) (Fig. 2).

#### Narrative framework

##### Temporal Coherence.

**Figure 3:**
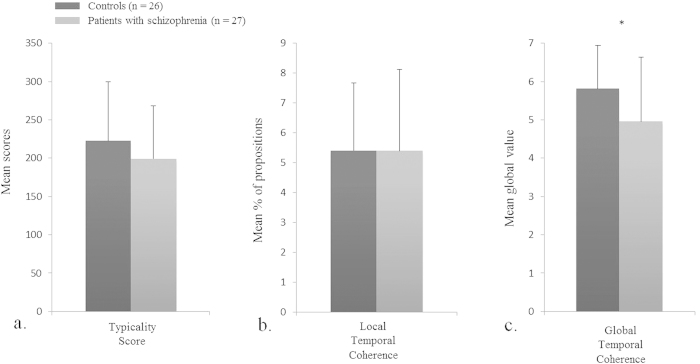
Narrative framework: typicality score of the cultural life script, and proportion of local indicators and global ratings of temporal coherence in patients with schizophrenia and controls. *p < .05.

The proportion of local temporal indicators did not differ significantly between groups (F(1,50) = 0.01; *p* = .92; ŋ^2^ = .00). The proportion of total anachronies was significantly higher in the patient group (M = 1.87; S.D. = 1.71) compared to control group (M = 1.11; S.D. = 0.66) (F(1,51) = 4.43; *p* = .04; ŋ^2^ = .08). Finally, global temporal coherence was significantly lower in patients’ life narratives (F(1,50) = 4.62; *p* = .04; ŋ^2^ = .08) (Fig. 3).

Cultural Life Script. The typicality of the cultural life script did not differ significantly between groups (F(1,47) = 1.27; *p=*.26; ŋ^2^ = .03) (Fig. 3).

### Correlational analyses

Correlations between local indicators and global coherence were found positive and significant in both groups for the temporal coherence, significant only in controls for causal-motivational coherence and not significant in either group for thematic coherence (see Table 2).

Concerning the centrality of chosen events, positive and significant correlations were observed between CES items in both groups, in particular items related to disruption were correlated with those related to integration (*rs* > .43; *n* = 26; *p* < .03) (see Supplementary Material S7).

In the patient group and at the local level, the proportion of both causal-motivational and thematic indicators of coherence (reflecting autobiographical reasoning) correlated with the global subjective sense of coherence (*r* = .41; *n* = 27; *p* = .03) and the correlation was even stronger with the subscale “meaningfulness” (*r* = .51; *n* = 27; *p* < .01). The averaged ratings of global causal-motivational and thematic coherence correlated positively with the shifting score (TMT B-A) (*r* = .40; *n* = 25; *p* = .047). Finally, measures of coherence did not correlate significantly with clinical symptoms in patients.

## Discussion

To the best of our knowledge, this is the first study which demonstrates that patients with schizophrenia are impaired in their ability to recount coherent narratives of their entire life. This study also provides an experimental assessment or objectification of what psychiatrists have been observing clinically for many decades, that is, patients displayed a dramatic reduction of autobiographical reasoning skills, which are essential to create and maintain coherence in life narratives despite autobiographical disruptions and changes. This impairment was correlated both with patients’ executive dysfunction and with the overall reduction of the subjective sense of coherence experienced by patients. Moreover, the narrative framework, which represents the basic scaffolding of the life story upon which major life events are ordered, was partially weakened in patients. These results cannot be explained by group differences in terms of IQ level, years of schooling, or of daily biographical practices of participants. Moreover, memories which were selected to structure the life story were rated as highly central for the self in both groups, which means that patients understood the procedure correctly. Finally, the reduced coherence of patients’ life narratives is apparently not due to a lack of the basic cultural knowledge which typically grounds the life story.

Regarding the narrative framework, our results showed that patients used as many temporal indicators as controls and that correlations between scores of local and global temporal coherence were significant in both groups. However, the global temporal coherence of patients’ narratives was high and yet significantly lower than that of controls’ narratives. Indeed, despite the presence of temporal indicators, anachronies were significantly increased in patients’ narratives, making it harder to understand the temporal location and order of events. This is consistent with previous studies showing that patients with schizophrenia narrated less chronologically ordered personal narratives than controls^24^. Taken together, our results show a weakness of the temporal component of the narrative framework of the life story despite a preservation of its cultural component.

As expected, causal-motivational and thematic coherence was dramatically reduced in almost all patients’ narratives. Patients with schizophrenia made fewer comments on changes that occurred throughout their life and to explain how these events have influenced the person they are today, which was reflected in a reduced global causal-motivational coherence. Our findings are in line with previous results showing that patients have difficulties to draw meanings from past personally significant events^16^,^17^,^44^. Contrary to controls, local indicators and global ratings of causal-motivational coherence were not significantly correlated in patients, suggesting that the impaired causal-motivational coherence of the entire life narrative of patients may involve other mechanisms than those involved in the ability to draw causal-motivational relationships with particular events.

In contrast, patients did not use fewer local indicators of thematic coherence than controls. In line with this result, one study^14^ showed that patients were able to give as many self-statements as controls to define themselves. However, thematic references were rare in our control group and we cannot rule out a possible floor effect that may have masked actual impairments in patients and a correlation between local indicators and global thematic coherence as found previously in healthy subjects^30^. In fact, when reading patients’ life narratives, the thematic guiding thread appeared to be more difficult to identify than in control narratives. Patients displayed difficulties interpreting events and integrating them along thematic lines, as confirmed by their lower score of global thematic coherence. These data confirm the reduction of thematic coherence that has been highlighted in narratives of Self-Defining Memories in patients with schizophrenia^44^. Moreover, it is worth mentioning that even if the proportion of self-statements was not significantly reduced in patients’ narratives, these self-statements are generally more uncertain and more changing over time than in controls^45^. Patients have difficulties articulating coherently different self-positions^12^. Taken together, this weakness of self-definition and self-representation across time may also account for the overall impairment of thematic coherence of patients’ narratives.

From the perspective of psychopathology, we argue that the reduced coherence of patients’ life narratives is related to disorders of the self in schizophrenia^40^. We suggest that the relationship between life story and self should be examined under the light of two complementary perspectives. First, the lack of life narrative coherence may point out the metacognitive dysfunction which is characteristic of schizophrenia^17^,^46^,^47^. Indeed, self-reflectivity deficiency may reduce patients’ ability to integrate different self-positions^12^ and to organize a coherent narrative of their life that establishes self-continuity and provides self-understanding of their own life^48^,^49^. Second, the reduced coherence of patients’ life narratives may also derive from an executive dysfunction, which is a core symptom of schizophrenia as well^50^. Executive functions play a critical role in the organization of autobiographical knowledge^8^, and our study showed a significant positive correlation between mental flexibility reduction and the decrease of coherence in patients’ narratives. Moreover, mental flexibility and the ability to draw lessons from past events have been shown impaired in schizophrenia^23^,^49^. While correlation analyses do not allow interpreting a causal direction of this association, one may consider that executive dysfunction may affect patients’ ability to construct a coherent life narrative and consequently alter, in the long run, the biographical and narrative representations of the self. In fact, these metacognitive and cognitive perspectives should be seen as complementary, because several studies have shown reciprocal relationships between metacognitive dysfunction and measures of executive functioning in schizophrenia^17^,^46^,^47^.

Based on our results, several questions remain unanswered. For instance, the lack of coherence of patients’ narratives may not actually derive from the impairment of autobiographical reasoning but could simply reflect the breakdown in life-continuity which follows the emergence of the illness in their life or mirror the disorganization of their daily life due to the illness. Against this hypothesis, our analyses of the CES items showed that life events chosen by the patients were neither more disruptive nor less integrated than those of controls, and that in both groups, the more disrupted an event is, the more integrated it is.

Our work has clinical implications because in addition to the objective impairments in life story coherence, patients reported a reduced subjective sense of coherence in their life. Indeed, patients experienced their environment as less coherent, predictable and explicable, and they reported lower meaning of their life. Interestingly, we found that patients with lower autobiographical reasoning skills reported weaker sense of coherence in their life. Previous studies have shown that autobiographical reasoning helps compensate effects of disruptions on the sense of self-continuity^28^, and that individuals with more coherent and well-integrated life story have a higher sense of self-continuity and self-understanding of their life^51^,^52^. Thus, patients’ difficulty to integrate changes in life into the self is reflected in their life narratives. This might represents one factor accounting for both the reduced sense of coherence reported by the patients and the reduced sense of self reported by clinicians.

Finally, our results advocate for the importance of therapeutic approaches such as narrative enhancement-based therapies to improve autobiographical reasoning skills in patients with schizophrenia^53^. These interventions have demonstrated positive changes on measures of well-being, lifestyle, cognitive skills, and self-experience in patients with severe mental disorders^11^,^54^,^55^,^56^ and may be recommended for reinforcing both self-experience and well-being^51^,^52^ in patients with schizophrenia.

## Additional Information

**How to cite this article**: Allé, M. C. *et al.* Impaired coherence of life narratives of patients with schizophrenia. *Sci. Rep.* **5**, 12934; doi: 10.1038/srep12934 (2015).

## Supplementary Material

Supplementary Information

## Figures and Tables

**Figure 1:**
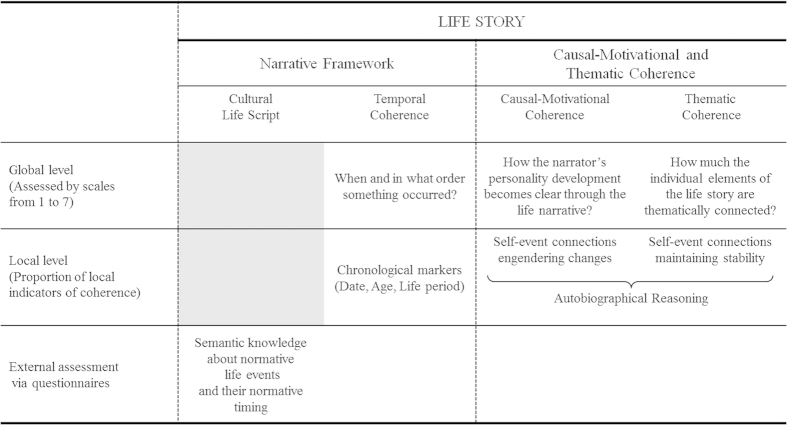
The four aspects of the life story and their methods of assessment (according to Köber *et al.*, 2015).

**Table 1:** Demographic, clinical and neuropsychological data of patients with schizophrenia and controls.

	Patients with schizophrenia(n = 27)		Controls(n = 26)		ANOVA		
	Mean	(SD)	Mean	(SD)	F	*p*-value	Effect size (ŋ^2^)
Age (years)	35.15	(8.91)	33.88	(9.80)	0.24	.62	0.05
Years of schooling	12.70	(2.37)	12.92	(2.40)	0.11	.74	0.02
BDI^a^			2.96	(3.38)			
CDSS^b^	1.67	(1.63)					
STAI-YA^c^	47.63	(7.25)	42.74	(7.06)	4.88	.03	0.11
STAI-YB^d^	49.58	(9.08)	39.04	(7.62)	16.73	<.001	0.29
Rosenberg Self-Esteem (RSE)	29.67	(4.52)	34.50	(3.83)	17.56	<.001	0.26
Onset of the illness (years)	22.00	(5.59)					
Duration of illness (years)	13.08	(8.28)					
PANSS^e^ (total score)	64.67	(19.37)					
- Postive symptoms	15.81	(5.37)					
- Negative symptoms	18.52	(6.81)					
- General symptoms	30.33	(10.05)					
f-NART^f^ (premorbid IQ^g^)	106.96	(8.34)	108.65	(4.74)	0.81	.37	0.02
WAIS-Rh (current IQ)	95.88	(13.59)	99.85	(8.56)	1.57	.22	0.03
Verbal fluency (z-score)	−0.09	(0.93)	0.63	(0.68)	10.11	.002	0.17
Shifting score (TMT^i^ B-A)	−0.42	(0.91)	0.13	(0.68)	5.91	.02	0.11
Biographical practices	31.74	(11.27)	31.27	(13.11)	0.02	.89	0.00
Sense of Coherence (total score)	127.04	(21.14)	160.08	(21.02)	32.54	<.001	0.39
- Manageability	43.52	(7.57)	55.11	(8.23)	28.54	<.001	0.36
- Meaningfullness	38.37	(6.43)	47.69	(6.47)	27.64	<.001	0.35
- Comprehensibility	45.15	(10.32)	57.27	(9.61)	19.53	<.001	0.28

^a^Beck Depression Inventory.

^b^Calgary Depression Scale for Schizophrenia.

^c^State-Trait Anxiety Inventory manual; part A assessing state anxiety.

^d^State-Trait Anxiety Inventory manual; part B assessing trait anxiety.

^e^Positive And Negative Syndrome Scale.

^f^French National Adult Reading Test.

^g^Intelligence Quotient.

^h^Wechsler Adult Intelligence Scale Revised.

^i^Trail-Making Test.

**Table 2:** Correlations between global ratings and local indicators of coherence in patients with schizophrenia and controls.

	Patients with schizophrenia (n = 27)			Controls (n = 26)		
	Global temporal coherence	Global causal-motivational coherence	Global thematic coherence	Global temporal coherence	Global causal-motivational coherence	Global thematic coherence
Local temporal coherence	0.45*			0.45*		
Local causal-motivational coherence		0.27			0.52**	
Local thematic coherence			0.32			−0.21

*p < .05; **p < .01.
