# Supplementary material for: Impaired coherence of life narratives of patients with schizophrenia
Source: Sci Rep. 2015 Aug 10;5:12934. doi: 10.1038/srep12934 (PMC4530446; doi:10.1038/srep12934)
Supplement: Supplementary Information [file srep12934-s1.pdf]

Impaired coherence of life narratives of patients with schizophrenia

Mélissa Allé[123], Jevita Potheegadoo[1234], Christin Köber[5], Priscille Schneider[346], Romain Coutelle[137], Tilmann Habermas[5], Jean-Marie Danion[1234], Fabrice Berna[1234*]

[1]INSERM U-1114, 1 place de l'Hôpital, Clinique Psychiatrique, Strasbourg Cedex, France

[2]Université de Strasbourg, Faculté de Médecine, 4 rue Kirchleger, Strasbourg, France

[3]FMTS: Fédération de Médecine Translationnelle de Strasbourg, France

[4]Hôpitaux Universitaires de Strasbourg, 1 place de l'Hôpital, Strasbourg Cedex, France

[5]Department of Psychology, Goethe University, Frankfurt am Main, Deutschland

[6]Centre Hospitalier de Rouffach, Centre de Ressources Autisme Alsace, 68250, Rouffach, France

[7]Centre Psychothérapique de Nancy, Centre de Ressources Autisme de Lorraine, 1 rue du Dr Archambault, BP 11010, Laxou, France

*Corresponding author:

Dr Fabrice Berna
Hôpitaux Universitaires de Strasbourg
1 place de l'Hôpital, Clinique Psychiatrique
67091 STRASBOURG CEDEX
FRANCE
Tel: +33(0)3-88-11-66-48 / Fax: +33(0)3-88-11-62-92
Email: fabrice.berna@chru-strasbourg.fr

Supplementary material

S1: Instructions for the seven most important memories

“First, I’d like to ask you to think about the seven most important events that have happened in your life. These may be events that have just happened, or they may have happened a long time ago. Then, please write your seven most important memories on these seven cards. Please name only memories of very specific events. Now please arrange these cards in the order in which the events happened on the table in front of you.”

S2: Instructions for life narratives

“Next, I’d like you to tell me a story involving your whole life. Please think about all the events that have happened in your life since you were born. Please integrate the seven events into your story. For example, you can tell me about the most important events in your life and the biggest changes. You can also tell me how what you have experienced is still important to you today and how it has influenced what kind of person you are today. Please take about 15-20 minutes to tell your story. I will not interrupt you. There is no right or wrong answer.”

S3: Example of segmentation into propositions, translated from a French narrative:

“So I was born with an English father and a French mother, / I was born in France, in Mulhouse, so in Alsace. / I spent my first two years with my mum only, / and when I was about two years, / my mum went / to join my father in England. / So I lived in England until I was 6.”

S4: Three Groups of Local Indicators of Global Coherence (according to Köber *et al.*, 2015)

Type of global coherence	Codes and Examples
Temporal	1) Temporal indicators: % of propositions<br>**Distance from present**: "I think this is half a year ago"; "2 years ago"<br>**Life phase:** "I got to know her in fourth grade"; "When I was a baby"<br>**Age:** "When I was 9"<br>**Calendar date:** "In 2002"; "On May 6, 2006"
Causal-Motivational	2) Self-event connections engendering changes: % of propositions<br>**Event explains change in personality:** "That journey changed many things for me; in that moment I understood what is meant by the meaning of life, and since then I am a little more self-confident."<br>**Events reveals unknown personality aspects:** "When I came back to Vietnam, I realized that in the meantime I had grown away from my own culture, the Vietnamese way of life, let's say from these Vietnamese traditional mentalities"<br><br>3) Autobiographical arguments: % of propositions<br>**Developmental status:** "At the time I wasn't aware of any of that, after all I was still too young for that."<br>**Biographical background:** "I really had problems with my teacher, she was my Physics teacher and today, out of defiance, I'm studying Physics."<br>**Formative experience:** "My burn-out has led me to no longer attach so much importance to money today"<br>**Learned lesson:** "After that I told myself, when I fall in love, then the next time I must, when I fall in love, take care that school doesn't suffer from that care that school doesn't suffer from that."<br>**Generalized Insight:** "I was missing him for many months. Probably it's always like that, when it's the first kiss."<br>**Turning points:** "The fact that all of a sudden the child was there turned my life upside down"
Thematic	4) Self-event connections maintaining stability: % of proportions<br>**Personality explains event, event is typical for personality:** "In puberty I was always extremely shy and well-behaved. I mean, I never rebelled against anything. So I was very restricted and limited in my ideas and possibilities. That's why I had never a boyfriend. I really was a late bloomer."<br>**Event is atypical for, or contradicts personality:** "Normally, I and the guys in my class, we are really uncool, I mean very well-behaving the whole time. But on that school trip, we freaked out. Oh man, I was so drunk."

S5: Three rating scales for global temporal, causal, and thematic coherence in life stories (according to Köber *et al*., 2015)

Type of global coherence	Descriptions: On three 7 point-scales, points 1-3-5-7 were used with the following descriptions. Points 2-4-6 were used in case of hesitations between two points.
Temporal	1) One can never tell when and in what order something occurred.
	2)
	3) One can often not tell when and in what order something occurred.
	4)
	5) One can mostly tell when and in what order something occurred.
	6)
	7) It is always crystal clear when and in what order something occurred.
Causal-Motivational	1) No development of the personality at all becomes clear.
	2)
	3) A development of the personality is described but not designated as such or still implicit.
	4)
	5) The development of the personality becomes clear and explicit on the whole through some of the events described.
	6)
	7) The development of the personality becomes clear and explicit in its turning-points and its motives.
Thematic	1) Between the individual episodes narrated no connection is discernible.
	2)
	3) With some episodes differing in content it is possible to recognize a common motive, theme or a thematic category but it is still implicit.
	4)
	5) There is an explicit attempt to establish a connection between episodes heterogeneous in content.
	6)
	7) Between the various heterogeneous episodes there is established a connection in a logical and comprehension fashion. A 7 is only given when a connection is also established between episodes about different areas of life.

S6: Memories' characteristics composing the life story of patients with schizophrenia and control participants

	Patients with schizophrenia (n = 27)		Controls (n = 26)		ANOVA		
	Mean	(SD)	Mean	(SD)	F	*p*-value	Effect size (η²)
Age at events occurrence	20.08	(5.55)	22.69	(7.42)	2.10	.15	0.04
Memory details*	5.40	(0.81)	5.87	(0.89)	3.95	.052	0.07
Emotional valence of memories*	4.46	(1.01)	5.24	(0.89)	8.89	.004	0.15
Emotional intensity of memories*	4.78	(1.06)	5.08	(0.95)	1.16	.29	0.02
Spectator/Active participant*	5.12	(1.3)	5.61	(0.82)	2.57	.11	0.05
Centrality of Event Scale (CES)**	23.36	(4.07)	24.65	(4.47)	1.20	.28	0.02
CES 1*[i] – I feel that this event has become part of my identity.	3.59	(0.68)	3.77	(0.67)	0.96	.33	0.02
CES 2*[i] – This event has become a reference point for the way I understand myself and the world.	3.26	(0.70)	3.50	(0.69)	1.55	.22	0.03
CES 3*[i] – I feel that this event has become a central part of my life story.	3.44	(0.74)	3.66	(0.67)	1.29	.26	0.02
CES 4*[I] – This event has colored the way I think and I feel about other experiences..	3.50	(0.62)	3.68	(0.66)	1.03	.31	0.02
CES 5*[d] – This event permanently changed my life.	3.14	(0.84)	3.47	(0.77)	2.22	.14	0.04
CES 6* - This event has become a reference point for the way I look upon my future.	2.80	(0.77)	2.90	(1.00)	0.16	.69	0.00
CES 7*[d] – This event was a turning point in my life.	3.62	(0.73)	3.66	(0.72)	0.05	.82	0.00

* range 1 to 7; ** range 7 to 49; [i] related to integration; [d] related to disruption

S7: Correlations between CES Items

a. In control group

	CES 1*[i]	CES 2*[i]	CES 3*[i]	CES 4*[i]	CES 5*[d]	CES 6*	CES 7*[d]
CES 1*[i]	-						
CES 2*[i]	.82***	-					
CES 3*[i]	.86***	.77***	-				
CES 4*[i]	.68***	.82***	.77***	-			
CES 5*[d]	.71***	.64***	.81***	.71***	-		
CES 6*	.59**	.54**	.63**	.59**	.73***	-	
CES 7*[d]	.62**	.64***	.66***	.74***	.84***	.75***	-

**p<.01; ***p<.001; [i] related to integration; [d] related to disruption

b. In patient group

	CES 1*[i]	CES 2*[i]	CES 3*[i]	CES 4*[i]	CES 5*[d]	CES 6*	CES 7*[d]
CES 1*[i]	-						
CES 2*[i]	.73***	-					
CES 3*[i]	.77***	.69***	-				
CES 4*[i]	.71***	.69***	.62**	-			
CES 5*[d]	.64***	.58**	.74***	.47*	-		
CES 6*	.28	.56**	.51**	.36	.44*	-	
CES 7*[d]	.58**	.54**	.65***	.45*	.76***	.43*	-

*p<.05; **p<.01; ***p<.001; [i] related to integration; [d] related to disruption
